# Emergence of Multiple SARS-CoV-2 Antibody Escape Variants in an Immunocompromised Host Undergoing Convalescent Plasma Treatment

**DOI:** 10.1128/mSphere.00480-21

**Published:** 2021-08-25

**Authors:** Liang Chen, Michael C. Zody, Clara Di Germanio, Rachel Martinelli, Jose R. Mediavilla, Marcus H. Cunningham, Kaelea Composto, Kar Fai Chow, Milena Kordalewska, André Corvelo, Dayna M. Oschwald, Samantha Fennessey, Marygrace Zetkulic, Sophia Dar, Yael Kramer, Barun Mathema, Soren Germer, Mars Stone, Graham Simmons, Michael P. Busch, Tom Maniatis, David S. Perlin, Barry N. Kreiswirth

**Affiliations:** a Hackensack Meridian Health Center for Discovery and Innovation, Nutley, New Jersey, USA; b Hackensack Meridian School of Medicine, Nutley, New Jersey, USA; c New York Genome Center, New York, New York, USA; d Vitalant Research Institute, San Francisco, California, USA; e Hackensack Medical University Center, Hackensack, New Jersey, USA; f Mailman School of Public Health, Columbia University Irving Medical Center, New York, New York, USA; University of Nebraska Medical Center

**Keywords:** SARS-CoV-2, convalescent plasma, immunosuppression, variants of concern, spike protein

## Abstract

Severe acute respiratory syndrome coronavirus 2 (SARS-CoV-2) variants of concern (VOCs), harboring spike protein N-terminal domain (NTD) or receptor-binding domain (RBD) mutations, exhibit reduced *in vitro* susceptibility to convalescent-phase serum, commercial antibody cocktails, and vaccine neutralization and have been associated with reinfections. The accumulation of these mutations could be the consequence of intrahost viral evolution due to prolonged infection in immunocompromised hosts. In this study, we document the microevolution of SARS-CoV-2 recovered from sequential tracheal aspirates from an immunosuppressed patient on steroids and convalescent plasma therapy and identify the emergence of multiple NTD and RBD mutations. SARS-CoV-2 genomes from the first swab (day 0) and from three tracheal aspirates (days 7, 21, and 27) were compared at the sequence level. We identified a mixed viral population with five different S protein mutations (141 to 144 deletion, 243 to 244 deletion, E484K, Q493K, and Q493R) at the NTD or RBD region from the second tracheal aspirate sample (day 21) and a predominance of the S protein 141 to 144 LGVY deletion and E484K mutant on day 27. The neutralizing antibodies against various S protein lentiviral pseudovirus mutants, as well as the anti-SARS-CoV-2 total Ig and IgG, showed “U” shape dynamics, in support of the endogenous development of neutralizing antibodies. The patient’s compromised immune status, the antirejection regiment, convalescent plasma treatment, and the development of neutralizing antibodies may have resulted in unique selective pressures on the intrahost genomic evolution, and this observation supports the hypotheses that VOCs can independently arise and that immunocompromised patients on convalescent plasma therapy are potential breeding grounds for immune escape mutants.

**IMPORTANCE** Over a year of the COVID-19 pandemic, distinct severe acute respiratory syndrome coronavirus 2 (SARS-CoV-2) lineages have arisen in multiple geographic areas around the world. SARS-CoV-2 variants of concern (VOCs), i.e., B.1.1.7 (alpha), B.1.351 (beta), P.1 (gamma), and B.1.617.2 (delta), harboring mutations and/or deletions in spike protein N-terminal domain (NTD) or receptor-binding domain (RBD) regions showed evidence of increased transmissibility and disease severity and possible reduced vaccine efficacy. In this study, we report the emergence of five different NTD and RBD mutations in an uncommon SARS-CoV-2 B.1.369 lineage from an immunosuppressed patient undergoing steroid and convalescent plasma therapy. The observation highlighted that VOCs can independently arise in immunocompromised populations undergoing anti-SARS-CoV-2 therapy, and enhanced measures will be required to reduce the transmission.

## INTRODUCTION

After a year of the COVID-19 pandemic, with >200 million global cases and 4 million deaths, the world is now focused on the biological consequences of the distribution of vaccines and the spread of “variants of concern” (VOCs). Four severe acute respiratory syndrome coronavirus 2 (SARS-CoV-2) VOCs, i.e., B.1.1.7 (20I/501Y.V1, alpha), B.1.351 (20H/501Y.V2, beta), P1 (20J/501Y.V3, gamma), and B.1.617.2 (delta), carrying the spike protein N501Y mutation emerged in the United Kingdom, South Africa, Brazil, Japan, and India (1–3) and have been associated with high transmissibility due to increased affinity to the angiotensin-converting enzyme 2 (ACE2) receptor. In each of these viruses, the spike protein contains clustered mutations in the N-terminal domain (NTD) and the receptor-binding domain (RBD) (e.g., E484K) regions. Some VOCs that carrying these mutations show reduced *in vitro* susceptibility to convalescent-phase serum, commercial monoclonal antibody cocktails, and vaccine neutralization and have been associated with increased rates of reinfection (4–7). The accumulation of these mutations is assumed to be the consequence of intrahost viral evolution, in part due to prolonged infection in immunocompromised hosts (8, 9). A recent report in the New England Journal of Medicine by Choi et al. (8) described the emergence of antibody escape mutations in an immunocompromised patient 75 days after infection. Here, we document the microevolution of SARS-CoV-2 recovered from sequential tracheal aspirates from an immunosuppressed patient on tacrolimus, steroid, and convalescent plasma therapy and identify the emergence of multiple NTD and RBD mutations associated with reduced antibody neutralization as early as 3 weeks after infection.

## RESULTS

### An immunocompromised COVID-19 patient.

**FIG 1:**
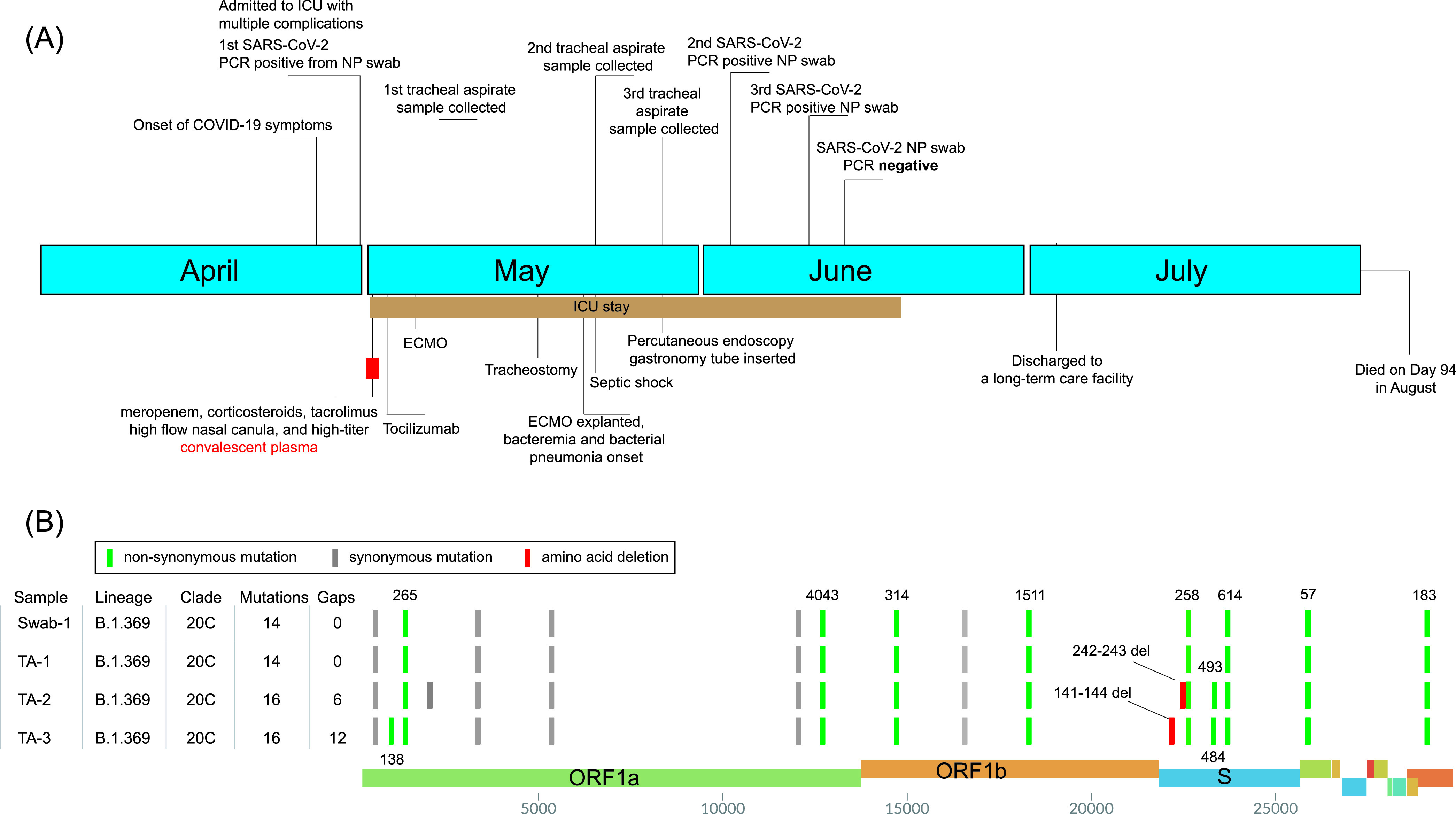
Clinical and genomic characterization of SARS-CoV-2 variations in an immunocompromised patient. (A) Clinical timeline of events of the immunocompromised patient. (B) SARS-CoV-2 genotypes of the major haplotypes from the swab (swab-1) and tracheal aspirate samples (TA-1, day 7; TA-2, day 21; and TA-3, day 27).

At the end of April 2020, a male in his early 50s was admitted in an intensive care unit (ICU) in a northern New Jersey hospital due to COVID-19 (Fig. 1A). He had a history of deceased donor kidney transplant for end-stage renal disease (ESRD) secondary to hypertension, complicated by cellular graft rejection and recurrent collapsing focal segmental glomerulosclerosis. He has been under immunosuppressive regimen of mycophenolic acid, prednisone, and tacrolimus along with multiple antihypertensive medications.

He was treated with high-titer convalescent plasma on day 1 of admission due to severe COVID-19 conditions and stayed in the ICU until day 49. His antihypertensives were discontinued due to his normotension, but his immunosuppressive regime was continued except for mycophenolate, given the likelihood of serious infection. Multiple nasopharyngeal swabs, tracheal aspirates, and serum samples were collected during his ICU stay (see below). A detailed patient history is described in Text S1 in the supplemental material.

10.1128/mSphere.00480-21.1 TEXT S1 Detailed case description. Download Text S1, DOCX file, 0.01 MB. Copyright © 2021 Chen et al. 2021 Chen et al. https://creativecommons.org/licenses/by/4.0/ This content is distributed under the terms of the Creative Commons Attribution 4.0 International license.

### Genomic analysis.

**FIG 2:**
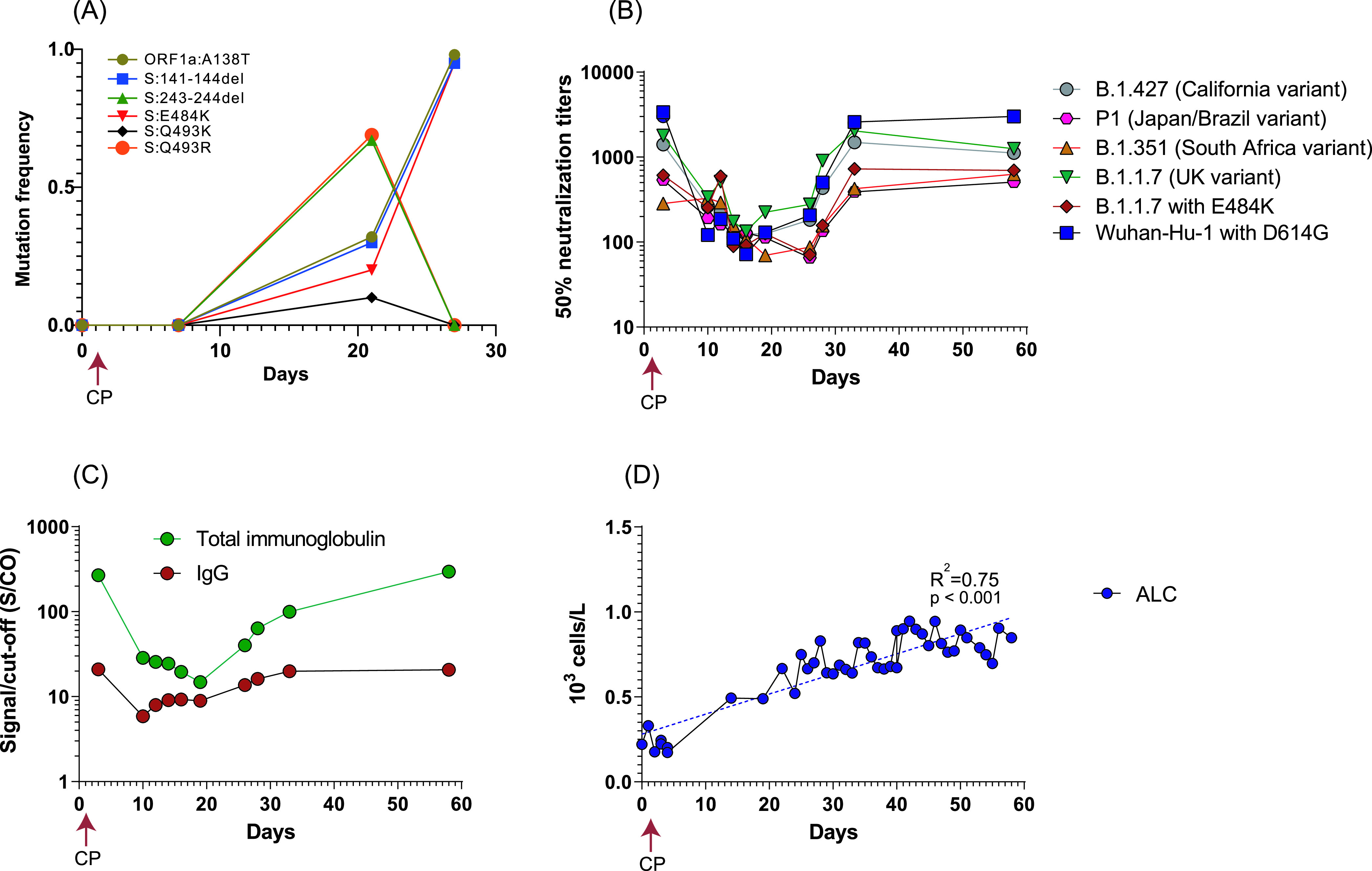
Dynamics of viral genomes, neutralization antibodies, anti-SARS-CoV-2 Ig/IgG, and absolute lymphocyte count (ALTs). (A) The mutation frequency changed among the swab and tracheal aspirate samples over time. (B) Fifty percent neutralization titers (NT_50_) of patient serum samples against PVs bearing different S protein variants. (C) Anti-SARS-CoV-2 Ig/IgG concentrations among the series of patient serum samples. (D) Change of patient ALTs overtime. CP, convalescent plasma.

SARS-CoV-2-positive quantitative reverse transcription-PCR (qRT-PCR) results (Table 1) were obtained from three nasopharyngeal swab samples (on days 0, 34, and 41) and three tracheal aspirates (on days 7, 21, and 27); the first swab and the three tracheal aspirates were available for viral genome sequencing (Fig. 1A and B). The genotypes of the initial swab and tracheal aspirate (day 7) were identical. The genomes of these two samples harbored 14 mutations (versus Wuhan-Hu-1) and were assigned Nextstrain clade 20C, Pangolin lineage B.1.369, and GISAID clade GH (Fig. 1B). Both AmpliSeq and total RNA sequencing (RNA-seq) analyses revealed that the second tracheal aspirate specimen (from day 21) had five different S protein mutations in the NTD or RBD region. The S protein Q493R substitution and 243 to 244 LA (243-244LA) deletion had ∼70% frequency, while open reading frame 1a (ORF1a) A138T, S protein 141 to 144 LGVY (141-144LGVY) deletion, and E484K and Q493K substitutions demonstrated ∼30%, ∼30%, ∼20%, and ∼10% mutation frequencies, respectively (Fig. 2A). However, the third tracheal aspirate sample collected 1 week later (day 27) was predominated by the haplotype ORF1a:A138T, S:141-144LGVY deletion, and S:E484K (>95% mutation frequency) (Fig. 2A).

**TABLE 1:** Cycle threshold values of SARS-CoV-2 samples

Sample	*C_T_* for N2 target
Swab-1 (day 0)	24.34
TA-1 (day 7)	20.43
TA-2 (day 21)	15.39
TA-3 (day 27)	24.65
Swab-2 (day 34)	37.14
Swab-3 (day 41)	32.39
Swab-4 (day 45)	Negative

The mixed viral population from the day 21 tracheal aspirate sample suggested the likelihood of in-host viral evolution or superinfection between days 7 and 21, as the viral genomes from the initial swab and day 7 tracheal aspirate samples were homogenous. If this was due to superinfection, the patient would have had to acquire three different viral genotypes, i.e., S:Q493R and 243-244LA, ORF1a:A138T, S:141-144del, and E484K, and ORF1a:A138T, S:141-144del, and S:E493K, circulating in the ICU at the same time. However, viral genome sequencing from more than 200 swab samples collected during the same time from the hospital failed to detect additional Pangolin lineage B.1.369 virus (data not shown), an observation in support of the likelihood of in-host evolution rather than superinfection.

The 141-144LGVY and 243-244LA deletions are located in the recently described “recurrent deletion regions” (RDRs) 2 and 4 (10), respectively, within the NTD of the spike protein. Deletions in the RDR region of the spike protein have been observed during prolonged infections in immunocompromised patients and proposed as a mechanism that evades the proofreading activity of the virus and accelerates adaptive evolution. The 141-144LGVY and 243-244LA deletions confer resistance to NTD-specific monoclonal antibody in neutralization assays (10). The Q493K/R and E484K substitutions are located in the RBD region of the spike protein and are associated with resistance to monoclonal antibodies or convalescent plasma (11, 12). In particular, the E484K mutation has been linked to the rapid spread of B.1.351 and B.1.1.28 variants in South Africa and Brazil, respectively. Intriguingly, the cooccurrence of 141-144LGVY and E484K in the third tracheal aspirate specimen completely replaced other mutants, suggesting this haplotype may have compensated for a fitness cost or have a higher antibody resistance level.

### Dynamics of SARS-CoV-2 antibody titers after convalescent plasma transfusion.

We next examined the SARS-CoV-2 neutralizing antibody (NAb) titers against variant S protein-bearing lentiviral pseudoviruses (PVs). Longitudinal serum samples collected from the patient on days 3, 10, 12, 14, 16, 19, 26, 28, 33, and 58 after admission were examined. The day 3 sample, which likely reflects the antibody titers from the convalescent plasma donor, demonstrated robust neutralization titers against all pseudoviruses, with the highest titers against Wuhan-Hu-1 with D614G PVs (>1:3,000) and medium levels against the B.1.1.7 (UK variant) and the B.1.427 (California variant) (1:1,400 to 1:1,800), but the lowest levels against variants harboring E484K mutations (B.1.1.7 with E484K, B.1.351, and P1; 1:250 to 1:350) (Fig. 2B). The NAb titers waned abruptly from day 3 to day 10 for all the PV variants and were maintained at low levels (∼1:100 to 1:200) until day 19. The declining NAbs may be partially explained by the waning neutralizing activity following the convalescent plasma transfusion. Interestingly, NAb titers for all the PV variants started to increase after day 19 and reached similar levels as that of day 3 at day 33 and day 56 (Fig. 2B).

The total anti-SARS-CoV-2 Ig and IgG concentrations showed patterns similar to those of the NAb titers (Fig. 2C). High titers of total Ig and IgG (268 and 21 signal to cutoff [S/CO], respectively, in comparison to previously reported median values of 101 and 11.7 from 370 convalescent plasma donors detected by the same assays) (13) were observed at day 3, followed by an abrupt reduction in S/CO values (∼90% total Ig and ∼70% IgG decrease, respectively) from day 3 to day 10, and then S/CO values were maintained at low levels until day 19. The S1 binding antibody S/CO values then started to increase and reached day 3 levels at day 58 (Fig. 2C). The increase of NAbs and S1 total Ig and IgG S/CO values aligned with the reduced viral loads (increased cycle threshold [*C_T_*] values) in the tracheal aspirates and nasopharyngeal swab samples (Table 1) as well as the emergence of antibody escape mutants (Fig. 2A). The “U” shape antibody titer dynamics also suggested the titer increase after day 19 most likely represents endogenous antibody production. Despite being under immunosuppressive treatment, the patient’s white blood cell (WBC) and neutrophil (ANC) counts were at 5.2 × 10^3^ ± 1.7 × 10^3^/μl (ranged from 3.5 × 10^3^ to 9.3 × 10^3^/μl) and 3.8 × 10^3^ ± 1.7 × 10^3^/μl (ranged from 1.7 × 10^3^ to 8.0 × 10^3^/μl), respectively, from day 0 to day 58 (data not shown). Although the absolute lymphocyte count (ALC) (<1 × 10^3^/μl during the stay) suggested the patient had lymphopenia, the ALC demonstrated steady improvement after convalescent plasma transfusion from day 0 (0.22 × 10^3^/μl) to day 58 (0.85 × 10^3^/μl) (*R*^2^ = 0.75, *P* < 0.001) (Fig. 2D).

## DISCUSSION

While most immunocompetent hosts are able to achieve resolution of COVID-19 within 1 to 3 weeks after symptom onset, there is emerging evidence that a preexisting immunocompromised state is associated with prolonged infection and significantly increased risk of severe disease (8, 9, 14, 15). Although the immunological mechanisms for control of SARS-CoV-2 in humans have not been fully elucidated, it is likely that both cytotoxic T cells and antibody-mediated immune responses are important for clearance of the viral infection (14, 16). In this study, this patient’s initial antirejection regimen of mycophenolate and tacrolimus targets and inhibits T-cell function and replication (17). While mycophenolate was discontinued, the patient was maintained on tacrolimus and prednisone during his entire hospitalization, which likely impaired his cellular immunity against SARS-CoV-2. Nevertheless, the patient developed a steady humoral immune response and generated NAbs, along with the increase of ALC. It is possible that the convalescent plasma transfusion given early in the disease course (day 1) partially neutralized the viral particles, allowing the humoral immune function to recover, supported by the increase of ALC from day 4 (Fig. 2D). The patient then started to endogenously develop antibodies, including NAbs (day 19), displaying higher titers against a nonescape virus (e.g., Wuhan-Hu-1) but lower titers against antibody escape variants harboring E484K (Fig. 2B).

In this study, multiple antibody escape mutants were detected in the tracheal aspirate samples. Potential factors contributing to the observed within-host evolution include the compromised immune status of the host, the antirejection regiment, and the passive (convalescent plasma-derived) and endogenously developed neutralizing antibodies, possibly resulting in a unique set of selective pressures compared with that in an immunocompetent host. These differential selective pressures could select for greater genetic diversity and reshape the dominant viral population throughout the course of infection. Notably, despite the emergence of multiple escape mutants, the patient developed antibodies and also showed low but robust neutralizing effects against the three VOC pseudoviruses (>1:500) (Fig. 2B) and eventually cleared the virus at day 45.

Taken together, our study suggests that differential selective pressure in an immunocompromised host could serve as the “breeding ground” for the emergence of immune escape mutants. Although we have no evidence that these escape variants were transmitted to others, this case supports growing evidence that VOCs may arise among immunocompromised populations undergoing anti-SARS-CoV-2 passive immunotherapy, and enhanced measures will be required to reduce transmission.

## MATERIALS AND METHODS

### SARS-CoV-2 detection.

Total nucleic acid (TNA) from nasopharyngeal swabs was extracted by the MagNAPure 24 system (Roche Life Science), and viral RNA from tracheal aspirates was extracted using QIAamp viral RNA minikit (Qiagen), according to the manufacturer’s instructions. SARS-CoV-2 detection was performed using our in-house developed and enhanced COVID-19 test (18), targeting SARS-CoV-2 E and N2 genes. The test was approved for use on 12 March 2020 under FDA Emergency Use Authorization for COVID-19 and has a limit of detection of less than 20 viral genome copies per reaction. A specimen is considered positive if the gene target has a cycle threshold (*C_T_*) value of <40.

### SARS‐CoV‐2 viral sequencing and genomic analysis.

SARS-CoV-2-targeted assay libraries were prepared using the AmpliSeq library Plus and cDNA synthesis for Illumina kits (Illumina) in accordance with manufacturer’s recommendations. Briefly, 20 ng of RNA was reverse transcribed followed by amplification of cDNA targets using the Illumina SARS-CoV-2 research panel (Illumina). The amplicons were then partially digested, ligated to AmpliSeq CD indexes, and then amplified using 18 cycles of PCR.

Total RNA sequencing libraries were prepared using the KAPA Hyper library preparation kit plus RiboErase, HMR (Roche), in accordance with manufacturer’s recommendations. Briefly, 50 to 200 ng of total RNA was used for ribosomal depletion and fragmentation. Depleted RNA underwent first- and second-strand cDNA synthesis followed by adenylation and ligation of unique dual-indexed adapters.

All libraries were quantified using fluorescent-based assays, including PicoGreen (Life Technologies), Qubit fluorometer (Invitrogen), and Fragment Analyzer (Advanced Analytics). Final libraries were sequenced on a NovaSeq 6000 sequencer (v1 chemistry) with 2× 150-bp reads. Two-duplicated AmpliSeq runs and one total RNA-seq run were performed for three tracheal aspirate samples. Two different AmpliSeq runs were performed, including one pooled with combinatorial indices that was run together with other nonviral samples and the other with each time point run in a lane containing no other viral samples. A summary of the viral genome sequencing statistics is provided in Table S1 in the supplemental material.

10.1128/mSphere.00480-21.2 TABLE S1 Characteristics of SARS-CoV-2 viral genome sequencing. Download Table S1, DOCX file, 0.01 MB. Copyright © 2021 Chen et al. 2021 Chen et al. https://creativecommons.org/licenses/by/4.0/ This content is distributed under the terms of the Creative Commons Attribution 4.0 International license.

Short-read data were filtered and processed prior to alignment. Read pairs that did not contain a single 19-bp seed k-mer in common with the SARS-CoV-2 genome reference (NC_045512.2) were discarded. Adapter sequences (AGATCGGAAGAGC and CTGTCTCTTATACACA) and low-quality (Q < 20) bases were trimmed from the remaining reads using Cutadapt v2.10 (19). After this, read pairs containing a mate shorter than 50 bp were removed. The remaining reads were then mapped to the SARS-CoV-2 genome reference using BWA-MEM v0.7.17 (20) with default parameters, and only read pairs with at least one alignment spanning a minimum of 42 bp in the reference and starting before position 29,862 (to exclude polyadenine-only alignments) were kept. Genome sequences were determined by alignment pileup consensus calling with a minimum support of 5 reads for total RNA and of 100 reads for AmpliSeq, using SAMtools v1.11 and bcftools v1.11 (21). Single nucleotide polymorphism (SNP) and indels were called using FreeBayes v1.3.5 (https://github.com/freebayes), followed by annotation using SnpEff v4.5 (22). A minimum variant calling frequency was set to be 5% to identify within-host variations.

The resulting SARS-CoV-2 viral genome sequences were uploaded to Nextclade server (https://clades.nextstrain.org/) to assign Nextstrain clades (23). SARS-CoV-2 lineage was determined using Pangolin v2.3.0 (https://github.com/cov-lineages/pangolin), and GISAID clade was determined based upon the clade-specific marker variants from https://www.gisaid.org (24).

### Anti-SARS-CoV-2 Ig/IgG and neutralizing antibody assays.

Anti‐SARS‐CoV‐2 total immunoglobulin (VITROS CoV2T) and IgG (VITROS CoV2G) testing was performed according to the manufacturer’s instructions (Ortho Clinical Diagnostics). The VITROS CoV2T test detects antibody to the S1 subunit of the SARS‐CoV‐2 spike glycoprotein, including IgA, IgM, and IgG, while CoV2G detects IgG antibodies to the same S1 antigen. The results were expressed as a signal-to-cutoff (S/CO) ratio. NAb titers were assessed using lentiviral pseudoviruses (PVs) bearing S proteins from different VOCs and encoding renilla luciferase (Integral Molecular). Briefly, serum was serially diluted and incubated with PVs for 1 h at 37°C before addition of 293T/ACE2 cells. After 3 days, cells were lysed and luciferase activity was measured.

### Clinical data collection and informed consent.

Informed consent was obtained from this patient, and the study was approved by Hackensack Meridian Health Institutional Review Board (IRB) under protocol Pro2018-1022. The clinical information and history, including the lab testing results, were extracted from electronic medical records.

### Data availability.

The consensus SARS-CoV-2 genome sequences from the four samples were deposited in GISAID (www.gisaid.org) under the accession numbers EPI_ISL_2193727, EPI_ISL_2224702, EPI_ISL_2224704, and EPI_ISL_2224707. Raw reads were deposited in GenBank under BioProject accession PRJNA675117.
