# Supplementary material for: Emergence of Multiple SARS-CoV-2 Antibody Escape Variants in an Immunocompromised Host Undergoing Convalescent Plasma Treatment
Source: mSphere. 2021 Aug 25;6(4):e00480-21. doi: 10.1128/mSphere.00480-21 (PMC8386433; doi:10.1128/mSphere.00480-21)
Supplement: TEXT S1 [file msphere.00480-21-t0001.docx]

**Detailed case description**

In the end of April 2020, a male in his early 50s presented to a Northern Jersey hospital with fever, productive cough, generalized myalgias, and progressive shortness of breath for 4 days (**Fig1A**). He had history of deceased donor kidney transplant for end-stage renal disease (ESRD) secondary to hypertension, complicated by cellular graft rejection and recurrent collapsing focal segmental glomerulosclerosis. On physical examination, the patient had tachypnea, a temperature of 102.3F and O2 saturation of 90% on 100% non-rebreather, but otherwise unremarkable. He was admitted under the suspicion of COVID-19 pneumonia. His medications were significant for his immunosuppressive regime of mycophenolic acid, prednisone, and tacrolimus along with multiple anti-hypertensive medications.

COVID-19 was confirmed to be positive by RT-PCR (Day 0). Chest X-Ray (CXR) revealed dense infiltrates bilaterally reflective of his viral pneumonia. Given his multiple comorbidities, immunosuppressed status, and labored breathing, he was admitted to the ICU for high flow oxygen and awake proning and was started on broad spectrum meropenem treatment. His anti-hypertensives were discontinued due to his normotension, but his immunosuppressive regime was continued except for mycophenolate given the likelihood of serious infection.

He was treated with high-titer convalescent plasma (Day 1) and tocilizumab (Day 2). Due to his worsening respiratory status, the patient was intubated (Day 2). Antibiotics were switched to vancomycin and piperacillin-tazobactam and then discontinued as the patient was afebrile (Day 3). The patient was found to have bilateral deep venous thrombosis and was started on therapeutic heparin (Day 3). Due to worsening hypoxic respiratory failure despite complete support from mechanical ventilation, the patient was subsequently cannulated and placed on veno-venous extra-corporeal oxygenation (ECMO) (Day 5). He went into rapid atrial flutter and was started on intravenous amiodarone (Day 5). His renal failure, attributed to multiple factors such as his tacrolimus, COVID-19 injury, and hypotension, slowly began to improve. Oxygenation began to improve and stabilize, leading to tracheostomy (Day 16) and ECMO explantation (Day 20). The patient, however, became febrile and septic with *Enterococcus* bacteremia and *Proteus mirabilis* pneumonia and he was restarted on vancomycin and piperacillin-tazobactam (Day 20). He subsequently developed septic shock and was started on vasopressors (Day 21). Following the antimicrobial susceptibility testing resutls, antibiotics were de-escalated to ampicillin (Day 21) and continued for a 7-day course. The septic shock resolved, and the patient was re-started on his anti-hypertensives once his blood pressure began to remain stable. His course continued to be complicated by periodic desaturations and wide and narrow complex tachycardia, anemia, and thrombocytopenia. He slowly improved permitting ventilation and sedation weaning. As his dysphagia was unresolved during his recovery, a percutaneous endoscopic gastrostomy (PEG) tube was placed (Day 28) to improve nutritional status. On day 45, his nasopharyngeal swab SARS-CoV-2 RT-PCR result turned out to be negative. He was transferred to the step-down unit as he continued to recover (Day 49) and was discharged to a long-term care facility (Day 64) requiring ventilatory support only at night. Unexpectedly, the patient expired presumably due to hypoxic respiratory failure secondary to his COVID-19 pneumonia (Day 94).
