# Supplementary material for: Emergence of Multiple SARS-CoV-2 Antibody Escape Variants in an Immunocompromised Host Undergoing Convalescent Plasma Treatment
Source: mSphere. 2021 Aug 25;6(4):e00480-21. doi: 10.1128/mSphere.00480-21 (PMC8386433; doi:10.1128/mSphere.00480-21)
Supplement: TABLE S1 [file msphere.00480-21-st001.docx]

**Table S1 Characteristics of SARS-CoV-2 viral genome sequencing**

|  | Total base (bp) | Total reads (n) | Mapping reads (n) | Mapping Rate (%) | Average sequence depth | Percent coverage  (%) |
| --- | --- | --- | --- | --- | --- | --- |
| TA-1-RNAseq | 107558100 | 717054 | 258094 | 35.99 | 1214 | >99.8% |
| TA-1-AmpliSeq-1 | 2289273000 | 15261820 | 15112551 | 99.02 | 6455 | >99.8% |
| TA-1-AmpliSeq-2 | 174050100 | 1160334 | 1149594 | 99.07 | 4607 | >99.8% |
| TA-2-RNAseq | 343586550 | 2290577 | 1474846 | 64.39 | 6485 | >99.8% |
| TA-2-AmpliSeq-1 | 720978600 | 4806524 | 4758612 | 99.00 | 6133 | >99.8% |
| TA-2-AmpliSeq-2 | 132147300 | 880982 | 879809 | 99.87 | 3917 | >99.8% |
| TA-3-RNAseq | 245054400 | 1633696 | 57944 | 3.55 | 44 | >99.8% |
| TA-3-AmpliSeq-1 | 1103325000 | 7355500 | 5957175 | 80.99 | 6197 | >99.8% |
| TA-3-AmpliSeq-2 | 343586550 | 2290577 | 1850948 | 80.81 | 5570 | >99.8% |

* RNASeq, the sequencing done by total RNAseq; AmpliSeq-1, the 1^st^ AmpliSeq run; Ampliseq-2, the 2^nd^ AmpliSseq run.
